# Modulation of the Activities of Catalase, Cu-Zn, Mn Superoxide Dismutase, and Glutathione Peroxidase in Adipocyte from Ovariectomised Female Rats with Metabolic Syndrome

**DOI:** 10.1155/2014/175080

**Published:** 2014-05-29

**Authors:** Rebeca Cambray Guerra, Alejandra Zuñiga-Muñoz, Verónica Guarner Lans, Eulises Díaz-Díaz, Carlos Alberto Tena Betancourt, Israel Pérez-Torres

**Affiliations:** ^1^Departments of Pathology, Instituto Nacional de Cardiología “Ignacio Chávez”, Juan Badiano 1, Sección XVI, Tlalpan, 14080 México, DF, Mexico; ^2^Departments of Physiology, Instituto Nacional de Cardiología “Ignacio Chávez”, Juan Badiano 1, Sección XVI, Tlalpan, 14080 México, DF, Mexico; ^3^Department of Reproductive Biology, Instituto Nacional de Ciencias Médicas y Nutrición “Salvador Zubirán”, Vasco de Quiroga 15, Sección XVI, Tlalpan, 14000 México, DF, Mexico; ^4^Departments of Vivarium, Instituto Nacional de Cardiología “Ignacio Chávez”, Juan Badiano 1, Sección XVI, Tlalpan, 14080 México, DF, Mexico

## Abstract

The aim of this study was to evaluate the association between estrogen removal, antioxidant enzymes, and oxidative stress generated by obesity in a MS female rat model. Thirty two female Wistar rats were divided into 4 groups: Control (C), MS, MS ovariectomized (Ovx), and MS Ovx plus estradiol (E_2_). MS was induced by administering 30% sucrose to drinking water for 24 weeks. After sacrifice, intra-abdominal fat was dissected; adipocytes were isolated and lipid peroxidation, non-enzymatic antioxidant capacity, and the activities of Cu-Zn and Mn superoxide dismutase (SOD), catalase (CAT), and glutathione peroxidase (GPx) were determined. There were no significant differences in the activities of Cu-Zn, Mn SOD, CAT, and GPx between the C and MS groups, but in the MS Ovx group there was a statistically significant decrease in the activities of these enzymes when compared to MS and MS Ovx+E_2_. The increased lipid peroxidation and nonenzymatic antioxidant capacity found in MS Ovx was significantly decreased when compared to MS and MS Ovx+E_2_. In conclusion, the removal of E_2_ by ovariectomy decreases the activity of the antioxidant enzymes in the intra-abdominal tissue of MS female rats; this is reflected by increased lipid peroxidation and decreased nonenzymatic antioxidant capacity.

## 1. Introduction

The metabolic syndrome (MS) is defined as a cluster of metabolic alterations [1], which includes the following diagnostic criteria: hypertension, diabetes, insulin resistance, dyslipidemia, and obesity [2]. Obesity, as component of MS, is considered a public health problem because of its magnitude and importance. Overweight and obesity are the fifth leading risk factor for death in the world [3]. The accumulation of abnormal or excessive fat stored in adipose tissue is the result of a chronic imbalance between energy intake and energy expenditure [2–4]. Adipose tissue, which was previously regarded as a tissue with few metabolic functions and considered only as passive reservoir, is now known to be metabolically active [5]. Several studies have suggested that obesity is associated with increased free radical concentrations [6], which can cause oxidative stress in the endoplasmic reticulum of the adipocyte [7]. An increase in oxygen consumption by mitochondria of the adipocyte [8] leads to excess processing of free fatty acids [9–11], which are produced by the hydrolysis of triglycerides in adipose tissue [12]. An excess of adipose tissue is also a source of inflammatory cytokines such as IL-1, IL-6, and TNF-*α*, and obesity is considered as a chronic inflammatory state. These cytokines are a potent stimulus for the production of reactive oxygen species [13]. Moreover, the mammalian cells are equipped with enzymatic antioxidant defense mechanisms among which are superoxide dismutase (SOD), catalase (CAT), glutathione peroxidase (GPx) enzyme, and the nonenzymatic systems such as vitamins A, C, and E, among others [14, 15]. The antioxidant enzymes contribute to eliminate radicals such as superoxide (O_2_^−^) and hydrogen peroxide (H_2_O_2_), preventing the formation of the very active species O_2_^−^ and radical hydroxyl (HO^−^) which are damaging to cells.

Sexual dimorphism is involved in the clinical manifestations of MS [16] and this disease tends to occur more often in postmenopausal than in premenopausal women [17–19]. Additionally, increased oxidative stress after menopause is associated with loss of endogenous estrogen synthesis [20]. Protection by female sex hormones is attributed to the well-demonstrated antioxidant properties of estrogen* in vivo*; estrogen decreases the occurrence of cardiovascular diseases in postmenopausal women [21], and* in vitro* estradiol acts as a molecule with antioxidant activity decreasing lipid peroxidation in rat liver microsomes [22]. GPx activity in female rat liver is increased by 60% when compared to Ovx female rats [23]. Behl et al. postulated that the antioxidant activity of estradiol in neuronal cells depends on the presence of the hydroxyl group (OH) at the C-3 position of the phenolic ring of the molecule [24], and another study showed that E_2_ can inhibit the oxidation cascades through the hydroxyl group of phenolic ring A [25]. Other antioxidant properties of estrogen action are exerted on glutathione (GSH). Cellular protection against oxidative stress has been demonstrated in neural cells, through the synergistic activity of estrogens and GSH [25]. Other studies show that postmenopausal women have a higher incidence of abdominal adiposity, associated with an increase in systemic levels of inflammatory cytokines, which suggests that estrogen can modulate body fat and systemic inflammation [26]. Stubbins et al. recently demonstrated that estrogen protected females against inflammation and oxidative stress when compared to males when studying intact female mice adipocytes, Ovx plus estradiol mice, and males subjected to a high-fat diet for 10 weeks [26]. It has been demonstrated that in kidney homogenates of intact rats with MS the activity of the enzymes CAT and SOD was significantly increased when compared to the MS Ovx group. These results suggest that female rats are protected against the estrogen prooxidant effects in the renal system induced by the high consumption of sucrose in the diet, but the protective effect decreases after ovariectomy [27].

On the other hand, Reaven and Ho [28] developed MS in rats by the administration of high-sucrose or fructose diets, which induce hypertriglyceridemia, hypertension, hyperinsulinemia, and insulin resistance and increase intra-abdominal fat tissue. In our laboratory we have developed a variant MS rat model by chronic administration of 30% sucrose in the drinking water for a period of 24 weeks.

Excess adipose tissue generates oxidative stress and estrogens have antioxidant properties; however, studies of the antioxidant capacity of estrogens in adipocytes are scarce. Therefore, the aim of this study was to evaluate the association between estrogen removal in MS female rat model and levels of antioxidant enzymes and oxidative stress generated by obesity.

## 2. Material and Methods

### 2.1. Animals

Experiments in animals were approved by the Laboratory Animal Care Committee of our institution and were conducted in compliance with the Guide for the Care and Use of Laboratory Animals of NIH. Weanling female rats weighing 100 ± 10 g, *n* = 8 per group. The groups were control (C), 30% sucrose-fed (MS), MS ovariectomized (Ovx), and MS Ovx + estradiol (E_2_). The animals were housed in ad hoc plastic boxes and were subjected to 12-hour light/obscurity cycles and environmental temperature between 18 and 26°C. They were fed commercial rodent pellets (23% of crude protein, 4.5% of crude fat, 8% of ashes, and 2.5% of added minerals; PMI Nutrition International, Inc., LabDiet 5008, Richmond, IN USA) ad libitum. At the end of the experimental period of 24 weeks, the rats were weighed and their blood pressure (BP) was measured by the tail-cuff method [27]; after overnight fasting, the animals were subjected to euthanasia with a guillotine and their blood was collected in vacutainer tubes. The samples were centrifuged for 20 min at 936 g and 4°C, in order to collect the serum in aliquots of 400 *μ*L and store it at −70°C.

### 2.2. Ovariectomy

Surgical ovariectomy was performed at 1 month of age. This was performed under anaesthesia (pentobarbital sodium 63 mg/Kg of body weight). The abdominal and pelvic area of the back was depilated, cleaned with soap, and disinfected with ethanol. A longitudinal incision of 1.5 cm was made, the skin was separated from the muscle, and a second incision of 0.5 cm was made in the muscle on both sides of the first, to exteriorize the ovaries. The Fallopian tubes were ligated and cut below the ligature. After the extirpation, the incision was sutured [27].

### 2.3. Hormonal Treatment

Estradiol valerate (Primogyn, Schering, Mexico; 1 mg/Kg body weight) was injected i.m. every 3 days, during the experimental period.

### 2.4. Measurement of Serum Sex Hormones

Serum estradiol was measured using the Diagnostic Products Corporation kit (Los Angeles, CA) and determination of some rat biochemical variables, such as glucose, cholesterol, triglycerides, and insulin, was determinate using commercially obtained kits. The HOMA index of resistance to insulin was calculated.

### 2.5. Isolation of Adipocytes

White adipocytes were isolated by collagenase digestion as described by Rodbell [29] with the following modifications: 4 g of adipose tissue was removed and transferred into Krebs buffer (containing 2% bovine serum albumin (BSA), 118 mM NaCl, 24 mM NaHCO_3_, 1.2 mM KH_2_PO_4_, 1.2 mM MgSO_4_, 4.7 mM KCl, 2.5 mM Ca_2_Cl, and 4.5 mM D-glucose at pH 7.35). Adipose tissue pieces were minced and digested with collagenase II (Sigma) and at 37°C for 90 min in a shaking water bath; the fat cell suspension thus obtained was filtered through a 250 *μ*m nylon mesh and centrifuged for 1 min at 300 g. The adipocytes collected from the top phase were washed with 10 ml of Krebs buffer without BSA three times, then resuspended in 900*μ*l buffer sucrose containing (1mM EDTA, 10 mM TRIS and 250 mM sucrose) and 100 *μ*L of protease inhibitors (1 mM PMSF, 2 mM pepstatin, 2 mM leupeptin, and 0.1% aprotinin) and homogenized; the sample was frozen at −30°C. Total proteins were determined by the method of Bradford [30].

### 2.6. Evaluation of Antioxidant Enzymatic System

The measurement of the activity of antioxidant enzymes was carried out by electrophoresis in 10% polyacrylamide native gels. To determine the activity of CAT, the gel was washed with distilled water during 5 minutes; this procedure was repeated three times; then it was incubated with a mixture of 1% K_3_Fe (CN)_6_ and 1% of FeCl_3_ 6H_2_O for 10 minutes in the dark and then washed with distilled water to stop the reaction [27].

To determine the activity of SOD, the gel was washed with distilled water during 5 minutes; this procedure was repeated three times; then it was incubated with 2.45 mM nitro blue tetrazolium (NBT) for 20 minutes; then the NBT solution was discarded and the gel was incubated in a solution of 28 mM EDTA, 0.028 mM riboflavin, and 36 mM phosphate buffer, pH 7.8. After 15 minutes of incubation in the dark, the NBT stain for O_2_ was developed by exposure to UV light for another 10 minutes [27]. The gels were analyzed by densitometry with an image Sigma Scan Pro 5 Analyzer.

The activity of enzyme GPX was measured by spectrophotometry: 1 mg of protein from the adipocyte homogenate was suspended in 1.6 mL of 50 mM phosphate buffer (pH 7.0), with added 0.2 mM NADPH, 1 mM GHS, and 1 UI/mL glutathione reductase. The mixture was incubated for 5 minutes at room temperature; then 100 *μ*L of 0.25 mM H_2_O_2_ was added and the reading was taken immediately at 340 nm (initial reading) and again after 5 minutes (final reading) [31].

### 2.7. Evaluation of Antioxidant Capacity of the Nonenzymatic System

For the precipitation of the proteins in the samples, 100 *μ*L of 10% ZnSO_4_ and 100 *μ*L of 0.5 N NaOH were added to one mg of protein from the adipocyte homogenate and centrifuged at 7155 ×g; the supernatant was suspended in 1.5 mL of reaction mixture (300 mM acetate buffer pH 3.6, 20 mM hexahydrate of ferric chloride, 10 mM of 2, 4, 6 Tris-2-pyridil-s-triazine dissolved in 40 mM chlorhydric acid (HCl) were added in a relation of 10 : 1 : 1 v/v, resp.); the mixture was shaken vigorously in vortex for 5 sec. Then it was incubated at 37°C for 15 minutes in the dark. The absorbance was measured at 593 nm. The calibration curve was obtained using *μ*mol Trolox equivalent [31].

### 2.8. Lipid Peroxidation (TBARS)

TBARS, a marker of damage by free radicals, was measured by a standard method. To 1 mg of protein from the adipocyte homogenate, 50 *μ*L methanol with 4% BHT plus phosphate buffer pH 7.4 was added. The mixture was shaken vigorously in a vortex for 5 seconds and then incubated in a water bath at 37°C for 30 minutes. This was followed by the addition of 1.5 mL of 0.8 M thiobarbituric acid and then incubated in a water bath at boiling temperature for 1 hour. After this time and to stop the reaction, the samples were placed on ice; 1 mL 5% KCL was added to each sample as well as 5 mL n-butanol; they were shaken in a vortex for 30 seconds and centrifuged at 2000 rpm., at room temperature for 2 minutes. Then the n-butanol phase was extracted and the absorbance was measured at 532 nm. The calibration curve was obtained using tetraethoxypropane as standard [27].

### 2.9. Statistical Analysis

Statistical analysis and graphics were performed with a SigmaPlot 11 program. The data are presented as the mean ± SEM. Statistical significance was determined by one-way ANOVA test, followed by the post hoc Tukey test. Differences were considered statistically significant at *P* < 0.05.

## 3. Results

### 3.1. General Characteristics

Table 1 shows some general and biochemical characteristics of the experimental animals. The body mass, intra-abdominal fat, triglycerides, insulin, and HOMA index were significantly elevated in MS in comparison with C (*P* < 0.001), and the MS Ovx increased it in comparison to MS intact (*P* < 0.01). Cholesterol and glucose remained at normal levels in all groups. In MS Ovx+E_2_ rats hormonal treatment did not modify the variables in comparison with MS intact rats.

### 3.2. Antioxidant Enzymes

**Figure 1:**
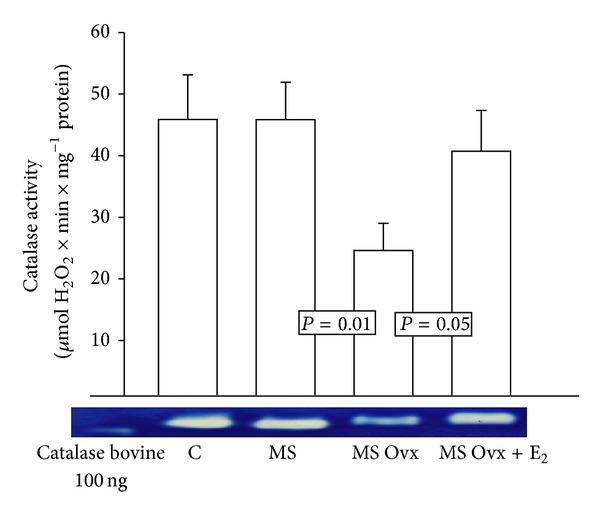
Effect of the estrogen removal and estradiol replacement on catalase activity in adipocyte homogenate. Native-gel electrophoresis with 10% polyacrylamide. CAT: catalase, C: control, MS: metabolic syndrome, MS Ovx: metabolic syndrome ovariectomized, and MS Ovx+E_2_: metabolic syndrome ovariectomized plus estradiol. Data are means ± SE; *n* = 8 in each group.

There was no difference in the activity of CAT in adipocyte homogenate between female C, MS intact, or MS Ovx+E_2_. However, a significant decrease in CAT activity was observed in MS Ovx when compared with MS intact (*P* = 0.01) (Figure 1). The treatment with estradiol induced a significant increase in MS Ovx+E_2_ versus MS Ovx (*P* = 0.05).

**Figure 2:**
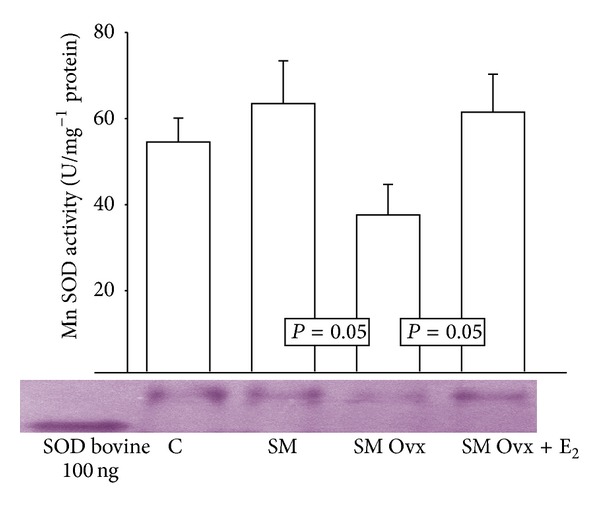
Effect of the estrogen removal and estradiol replacement on Mn SOD activity in adipocyte homogenate. Mn SOD: superoxide dismutase manganese. Native-gel electrophoresis with 10% polyacrylamide. Data are means ± SE; *n* = 8 in each group.

**Figure 3:**
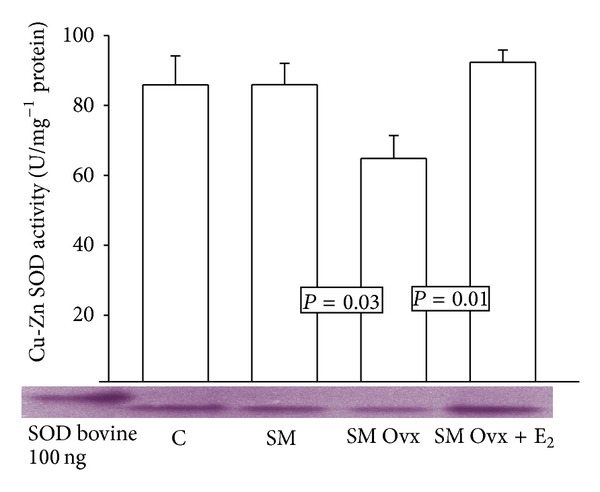
Cu-Zn SOD activity in adipocyte homogenate of experimental groups. Cu-Zn SOD: superoxide dismutase copper-zinc. Data are means ± SE; *n* = 8 in each group.

There were no differences in Mn SOD activity except between MS intact and MS Ovx, the latter being reduced (*P* = 0.05) (Figure 2), but treatment with estradiol significantly increased it in MS Ovx+E_2_ versus MS Ovx (*P* = 0.05).  Figure 3 shows the Cu-Zn SOD activity. There were no significant differences between groups C, MS intact, or MS Ovx+E_2_. However, a significant decrease in this enzyme activity was observed in MS Ovx when compared with MS intact (*P* = 0.03) (Figure 1). The treatment with estradiol induced a significant increase in MS Ovx+E_2_ versus MS Ovx (*P* = 0.01).

**Figure 4:**
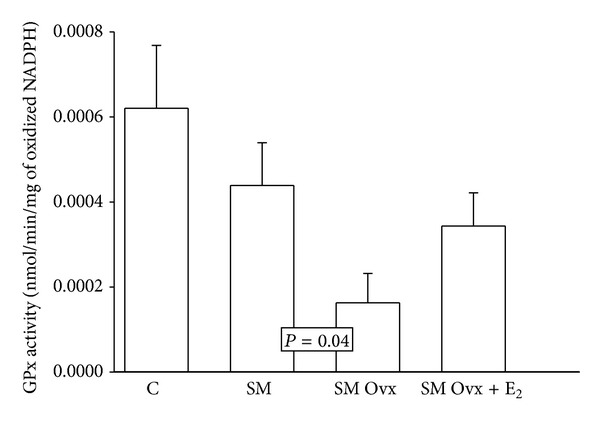
Effect of the ovariectomy and estradiol replacement on glutathione peroxidase activity in the adipocyte homogenate. Data are means ± SE; *n* = 8 each group.

Figure 4 shows the activity of the GPx enzyme in C and MS intact groups; there are no significant changes; however, the MS Ovx group showed lower activity than the MS intact rat group (*P* = 0.04). The treatment with estradiol in MS Ovx+E_2_ rats group tended to increase the activity but the change was not statistically significant.

### 3.3. Lipid Peroxidation (TBARS)

**Figure 5:**
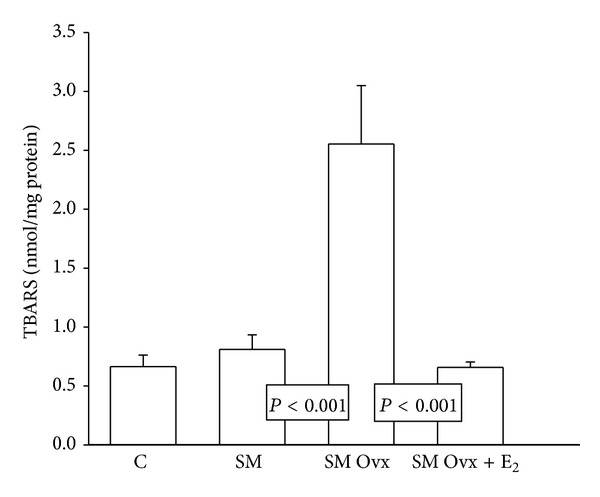
Lipid peroxidation was measured in adipocyte homogenate. See Table 1 legend for abbreviations. Values are means ± SE; *n* = 8 each group.

The lipid peroxidation index in adipocyte homogenates showed no changes between C and MS intact. Ovariectomy increased lipid peroxidation in the MS group in comparison to MS intact and MS Ovx+E_2_ rats groups (*P* < 0.001). The hormonal treatment did not produce significant changes in MS Ovx+E_2_ when compared to MS intact group (Figure 5).

### 3.4. Antioxidant Capacity of the Nonenzymatic System

**Figure 6:**
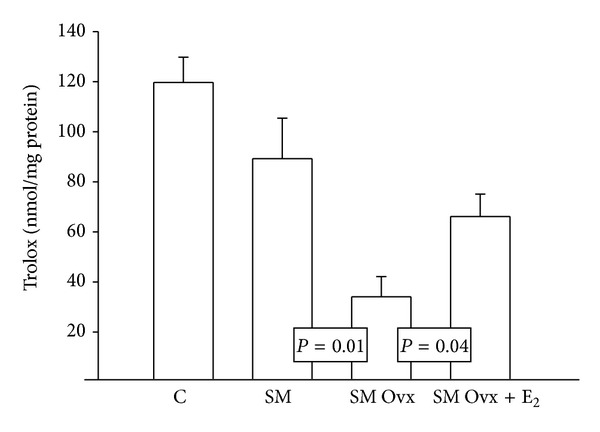
Effects of the ovariectomy and estradiol replacement upon the antioxidant capacity of the nonenzymatic system. See Table 1 legend for abbreviations. Values are means ± SE; *n* = 8 each group.

Figure 6 shows that there were no statistically significant changes in the antioxidant capacity of the nonenzymatic system in the C and MS intact group but ovariectomy decreased the antioxidant capacity in the MS Ovx group versus MS intact group (*P* = 0.01). In the MS Ovx+E_2_ group there were significant increases in the antioxidant capacity of the nonenzymatic system in comparison with MS Ovx (*P* = 0.04).

## 4. Discussion

Obesity is a component of MS and is the consequence result of a positive energy balance resulting from the interaction of several factors, including feeding, reduced physical activity, and genetic components. There is enough literature showing that in diseased states, such as MS and obesity, there is increased systemic oxidative stress [32–34]. Moreover, the reduction of the synthesis endogenous estrogen is associated with the onset of MS development. The aim of this study was to evaluate the association between estrogen removal and antioxidant enzymes and oxidative stress generated by obesity in a MS female rat model.

### 4.1. Body Weight and Intra-Abdominal Fat

The results showed that body weight in the MS group was significantly higher than in the C group; however, the group with the highest increase in weight was the MS Ovx group; these results are similar to those obtained by Stubbins et al. who showed that after 10 weeks of consuming high-fat diet male mice had significantly higher body weight than intact female mice. Ovx females show similar changes to male mice with respect to changes in body weight, but when supplemented with estradiol, changes in body weight were minimal and similar to intact females [26]. Accumulation of intra-abdominal adipose tissue is considered as a risk factor for the development of MS [35–37]. Intra-abdominal fat is more expandable than subcutaneous adipose tissue; it is metabolically active and secretes substances directly to the portal circulation such as inflammatory cytokines and free fatty acids which are associated with insulin resistance, hypertension, and cardiometabolic risk [38]. Estrogens are important regulators of adipose tissue deposition in humans, rodents, and other species [39]. Our results show that MS Ovx group had a higher amount of intra-abdominal fat when compared to MS intact and MS Ovx+E_2_ groups. These results suggest that intra-abdominal fat increases in the MS Ovx group and this is probably due to the absence of estradiol. In premenopausal women it has been reported that fat tissue is located primarily in subcutaneous deposits; however, at menopause, there is redistribution to visceral adiposity, which is sensitive to estrogen therapy [40]. It has been described that the intra-abdominal adipose tissue expresses *α* and *β* receptors but that *α* receptor expression is predominant; Meyer et al. have reported that female mice lacking *α* estrogen receptor develop central obesity [40]. Likewise, Brown et al. mention that estrogens can regulate energy intake through direct action of *α* estrogen receptor or through indirect action decreasing orexigenic peptides; therefore the absence of estrogens may promote hyperphagia, although some authors report that ovariectomy is not necessarily accompanied by increased food intake [35]. Another study, in a female mice model subjected to ovariectomy and in which there was no decrease in energy expenditure or concomitant changes in energy intake and adipocyte hypertrophy, showed that Ovx female mice replaced with estradiol were protected from adipocyte hypertrophy [36]. Estrogen may directly inhibit the deposition of adipose tissue by reducing lipogenesis through decreased mRNA, activity, and expression of lipoprotein lipase, an enzyme that regulates the storage of triglycerides in the adipocytes [39]. It has been described that ovariectomy can increase the activity of lipoprotein lipase and lipid deposition in adipocytes, but the administration of physiological estrogen doses reverses this condition. Our results show that the MS Ovx rats had increased body weight and intra-abdominal adipose tissue when compared to the MS intact and MS Ovx+E_2_ rats. In addition, estrogens may also affect lipid deposition through the hormone-sensitive lipase and increased fatty acid oxidation, diminishing the likelihood of lipotoxicity [41]. It has been reported that in postmenopausal women the adipocyte hypertrophy and lipolytic activity are high, which may explain why postmenopausal women show high levels systemic of FFA [26, 42].

### 4.2. Systolic Blood Pressure

The MS Ovx group showed a significant increase in SBP when compared to the other groups. This result is consistent with basic and clinical research, in which it was demonstrated that the SBP is elevated in premenopausal women compared to postmenopausal women [43, 44]. In addition, it is known that nitric oxide, a potent vasodilator, participates in the regulation of SBP [27]; nitric oxide metabolism is better preserved in females than in males, partly as a result of the action of estrogen [44]. The results show that, in the C groups and MS and MS Ovx+E_2_ groups, SBP was decreased when compared to MS Ovx group. Another study showed that estradiol replacement in MS Ovx female rats with MS is associated with an increased activity of nitric oxide synthase, endothelium-dependent vasodilation, and decreased blood pressure [45].

### 4.3. Hypertriglyceridemia

Diets high in carbohydrates, such as fructose, sucrose, or both, induce hypertriglyceridemia and reduction in antioxidant reserves [45]. The results show that triglycerides are increased in MS groups and decreased when compared to the C group and are similar to those obtained by Pettersson et al. who induced MS in female rats by administering a high-fat diet (60%) over a period of 14 weeks and found a significant increase in triglycerides when compared to the C group [46]. Another study showed that hypertriglyceridemia, resulting from the intake of high carbohydrate diet in rats, was associated with hyperinsulinemia, increased SBP, and insulin resistance [47]. Moreover, decreased levels of ovarian hormones associated with menopause or ovariectomy have been related to a decrease in glucose uptake by insulin [48]. Our results show that the serum insulin levels were increased in the MS group when compared to the C group, but the group MS Ovx showed the highest values; therefore, it appears that estrogen may have protective effect against the development of hyperinsulinemia.

### 4.4. Insulin Resistance

In addition, alterations in lipid metabolism and fat body distribution coupled with estrogen deficiency have been postulated as a causative factor contributing to the increased prevalence of insulin resistance in postmenopausal when compared to premenopausal women [48]. Furthermore, as previously mentioned, the MS Ovx group was the one that had the highest rate of insulin resistance when compared to the MS Ovx+E_2_. Saglam et al. showed that hormone replacement therapy increased insulin peripheral action in postmenopausal women [49], and, in another study, Abbas and Elsamanoudy found that estrogen exerted effects upon insulin resistance; these authors found that the estrogen administration in Ovx rats significantly decreased the plasma glucose, insulin concentration, and HOMA index when compared with C [48]. Insulin binding to its receptors on the cell membrane is required to cause the hormonal actions. Therefore, the structure and functional integrity of the cell membrane influence the properties of the insulin receptor. Cell membrane properties, particularly its fluidity, depend upon the fatty acid composition. In hyperinsulinemic states, increased saturated fatty acids lead to a decrease in the affinity and number of insulin receptors, which may cause insulin resistance associated with hyperinsulinemia [47]. Estrogens can improve insulin action by increasing receptor specific binding to insulin. Evidence suggests that estrogen can increase the action of insulin in adipocytes via activation of transcription factors (protein-1 activator than response to cAMP) which are orchestrated by insulin [35, 48]. Moreover, a high concentration of H_2_O_2_ (100 *μ*M) decreases insulin receptor affinity [50]. Our results show that lipoperoxidation index in MS Ovx group was higher when compared to the other experimental groups; this suggests that the elimination of estradiol through ovariectomy increases reactive oxygen species that can oxidize polyunsaturated fatty acids of cell membrane favoring insulin resistance and oxidative stress.

### 4.5. Leptin

Leptin, an adipokine secreted by adipose tissue [51], is directly proportional to the fat content [52]. The results show that the ovariectomy reduces serum leptin concentration in comparison to MS intact female rats and this was restored by estradiol replacement. These results suggest that estradiol may modulate leptin secreted by adipocytes. To support the above, it has been described that leptin levels are increased in women when compared to men, partly as result of inhibition of androgen and estrogen stimulation [51]. Leptin action is mediated via a specific receptor Ob-Rb, located mainly in the hypothalamus; estrogen may modulate the catabolic action of leptin in the brain and it has been described that estrogens are associated with increased leptin sensitivity [35]. A study by Alonso et al. demonstrated that estradiol can directly modulate the expression of Ob-Rb receptor in adipose tissue and skeletal muscle [53]. Another study in Ovx rats demonstrated that leptin mRNA expression in adipose tissue was associated with decreased plasma leptin concentration when compared to rats treated with estradiol. The mechanism responsible for the effect of estrogen on leptin concentrations in plasma remains unclear, but it is postulated that estrogen may have a direct action on the leptin gene in the adipocytes [53].

### 4.6. Antioxidant Enzymes

Investigations on the differences in gender related antioxidant reserves include studies in humans and in several animal species in both normal populations and pathological conditions and different organs. Several studies indicate that females have higher antioxidant potential, given by the enzymatic and nonenzymatic activity [54]. In addition, the activity of the antioxidant enzymes SOD, Cat, and GPx plays an important role in obesity associated with MS. The results show that the activity of the Cu-Zn SOD in the MS Ovx group was significantly decreased when compared to the MS intact and MS Ovx+E_2_ group; concerning the activity of Mn SOD, the results showed the same tendency. This suggests that estradiol may modulate the activity of both SOD isoforms. It has been described that estrogens may regulate the nuclear transcription factor, Nrf-2 pathway, which controls the expression and induction of genes that are encoded for phase II antioxidant enzymes, including SOD isoforms [55]. Moreover, our results are similar to those of a study conducted by Kumar et al., in which the antioxidant effect of estradiol in liver fractions of rat females of 3, 12, and 24 months of age was evaluated which showed that the treatment with estrogens normalized the decrease of the SOD activity induced by aging and menopause [56]. Another study in kidney homogenate of MS female rat, which evaluated the activity of the Cu-Zn and Mn SOD, with estradiol replacement, showed an increase in the activity of these enzymes in the control rats in comparison to MS rats; the activity decreased after ovariectomy and treatment with E_2_ restored it [27]. Furthermore, Baños et al. showed that SOD activity in the heart of MS male rats was decreased while oxidative stress was increased but these changes were not present in female rats [54]. Busserolles et al. demonstrated that the SOD activity was decreased in the heart of male rats that were fed with sucrose for two weeks, when compared to female rats [57]. Moreover, it has been shown that fructose or sucrose may inactivate CAT* in vitro *[58]. However, our results on CAT activity in adipocyte homogenates showed no significant changes between C, MS, and MS Ovx+E_2_ groups but showed significant difference when comparing the MS intact and Ovx+E_2_ group with the MS Ovx group. These results are similar to those of other studies which showed significant decrease in CAT activity in the MS Ovx female rats when compared to MS female intact and replaced with estradiol [27]. Therefore, the results suggest that E_2_ can promote an increase in CAT activity. Furthermore, Pajović and Saicić mention that females had lower oxidative stress in the brain and increased activity of CAT in comparison with males [59]. Regarding the activity of GPx, the results show that the control group showed no significant difference when compared to the MS group, but there was significant decrease in the activity of this enzyme in the MS Ovx group compared to MS intact group. Baltgalvis et al. demonstrated in murine skeletal muscle that genes encoded for type 3 GPx expressions are sensitive to estradiol and regulated via *α* receptors [60]. In addition, another study showed that the activity of GPx was significantly increased in premenopausal women and it decreased after menopause [61]. However, another study showed no significant difference in the activity of this enzyme in the brain of male and female mice, while in the liver the enzyme activity was significantly higher in females than in males [59]. Supporting the above,* in vitro* studies have shown that damage induced in myocardial cells by free radicals was stopped by nuclear translocation of Nrf-2 and that this effect was promoted by pretreatment with estrogen, since it controls and induces expression of genes encoded for GPx [55]. Likewise, other investigations have shown synergistic interaction between estrogens and GSH in neuronal protection against oxidative stress [25].

### 4.7. Nonenzymatic Antioxidant Capacity

The nonenzymatic antioxidant capacity in adipocyte homogenates showed no significant differences when comparing the C group with the MS group, but the MS Ovx group showed a significant decrease in comparison with MS intact and MS Ovx+E_2_. This result suggests that the E_2_ have antioxidant properties that allow the increase in nonenzymatic antioxidant capacity in the adipocytes of the MS rats. Another study, which determined the protection of estradiol upon oxidative stress in visceral tissue in a murine model subjected to high-fat diet, showed that male mice and Ovx females have a significant increase in *γ*H2AX, a biomarker for oxidative stress, in adipocyte core compared to intact and Ovx females with estradiol replacement. The researchers concluded that estradiol may protect from the development of oxidative stress in adipose tissue [26].

### 4.8. Lipid Peroxidation (TBARS)

With respect to lipid peroxidation, the results show no significant difference in the C group compared to the MS groups, but in the MS Ovx group there was a significant increase when compared to MS and MS Ovx+E_2_ group, which had similar lipoperoxidation values. Moreover, a study conducted by Taskiran and Evren demonstrated that H_2_O_2_ induced lipid peroxidation in cell cultures of adipose tissue and that it was attenuated with pretreatment with different concentrations of estradiol; the maximum effect was observed at 10 nM [62]. Signorelli et al. demonstrated that oxidative stress damage, measured by the concentrations of 4-hydroxynonenal, a waste product of the oxidation of lipids, was significantly increased in postmenopausal women when compared to premenopausal women, which suggest that estrogen may protect against lipid peroxidation [61]. The effect of estrogen decreasing the rate of lipid peroxidation can be explained by the key structure of the phenolic ring of estradiol which renders the molecule with antioxidant protection [63]. Wang et al. proposed that cell membranes are one of the primary targets of the antioxidant effects of estrogen. Antioxidant actions of estrogens on cell membranes are independent of estrogen receptor and the phenolic ring structure may play an important role in this effect [25]. In addition, another study postulated that the antioxidant capacity of E_2_ in neuronal cells depends on the presence of the hydroxyl group at the C-3 position of the phenolic ring of the molecule [24]. This mechanism has also been previously demonstrated by Jellinck and Bradlow who postulated that E_2_ could inhibit oxidative cascades through the hydroxyl group of the A phenolic ring [25, 64].

## 5. Conclusions

In conclusion, the results suggest that removal of E_2_ by ovariectomy decreases the activity of the antioxidant enzymes in the intra-abdominal tissue of MS female rats; this is reflected in increased lipid peroxidation and decreased nonenzymatic antioxidant capacity. Replacement with E_2_ can protect MS female rats from increases in body weight, intra-abdominal fat accumulation, and hypertension. It can also improve insulin sensitivity by decreasing insulin resistance and sensitivity to leptin. The oxidative stress and obesity present in MS ovariectomized female rats may be attenuated by hormonal replacement therapy; however, more studies are still needed on the antioxidant capacity of E_2_ in metabolic syndrome.

## Figures and Tables

**Table 1:** General and biochemical characteristics.

Variables	C	SM	SM Ovx	SM Ovx+E_2_
Body mass (g)	272.5 ± 6.2	305.4 ± 2.0**	377.4 ± 2.6^††^	296.2 ± 3.2
Intra-abdominal fat (g)	4.8 ± 0.5	7.1 ± 0.4*	13.3 ± 0.7^††^	6.0 ± 0.1
Systolic blood pressure (mm/Hg)	116.6 ± 2.6	125.3 ± 3.1	146.1 ± 2.2^††^	124.5 ± 1.8
Cholesterol (mg/dL^−1^)	68.2 ± 3.3	69.2 ± 5.0	69.7 ± 2.8	70.0 ± 3.6
Triglycerides (mg/dL^−1^)	57.2 ± 5.8	96.2 ± 10.0**	105.5 ± 5.9	108.7 ± 9.1
Glucose (mmol/dL^−1^)	6.4 ± 0.2	6.3 ± 0.3	7.3 ± 0.4	6.3 ± 0.2
Insulin (*μ*UI/mL^−1^)	2.7 ± 0.7	7.6 ± 0.9**	11.6 ± 1.5^†^	6.1 ± 1.2
HOMA	0.5 ± 0.1	2.5 ± 0.4*	3.9 ± 0.6^†^	1.8 ± 0.3
Leptin (ng/mL)	3.2 ± 0.5	2.8 ± 0.4	1.3 ± 0.1^†^	2.1 ± 0.2
Estradiol (pg/mL)	24.8 ± 3.2	29.1 ± 5.8	9.2 ± 1.1^†^	26.8 ± 3.6

Data are means ± SE; *n* = 8 each group. Statistically significant at C versus MS *P < 0.01 and **P < 0.001; ^†^ *P* < 0.01 and ^††^ *P* < 0.001 MS versus MS Ovx. C: control; MS: metabolic syndrome; MS Ovx: metabolic syndrome ovariectomized; MS Ovx+E_2_: metabolic syndrome ovariectomized plus estradiol.
